# The Long and Winding Road to Understanding Autism

**DOI:** 10.3390/neurosci6030084

**Published:** 2025-09-03

**Authors:** Jorge Manzo, María Elena Hernández-Aguilar, María Rebeca Toledo-Cárdenas, Deissy Herrera-Covarrubias, Genaro A. Coria-Avila, Hugo M. Libreros-Jiménez, Lauro Fernández-Cañedo, Lizbeth A. Ortega-Pineda

**Affiliations:** 1 Instituto de Investigaciones Cerebrales, Universidad Veracruzana, Xalapa 91190, Ver., Mexico; elenahernandez@uv.mx (M.E.H.-A.); rtoledo@uv.mx (M.R.T.-C.); dherrera@uv.mx (D.H.-C.); gcoria@uv.mx (G.A.C.-A.); hlibrerosjimenez@arizona.edu (H.M.L.-J.); 2 Department of Biomedical Engineering, University of Arizona, Tucson, AZ 85721, USA; 3 Facultad de Medicina, Universidad Veracruzana, Xalapa 91017, Ver., Mexico; lafernandez@uv.mx (L.F.-C.); liortega@uv.mx (L.A.O.-P.)

**Keywords:** Autism Spectrum Disorder, neurodevelopment, Autism history

## Abstract

Autism Spectrum Disorder presents one of the most complex challenges in contemporary neuroscience. This review adopts an unconventional narrative structure, drawing inspiration from song titles by The Beatles to explore the multifaceted biological, developmental, and social dimensions of autism. Spanning historical perspectives to embryonic origins and adult cognition, we examine critical topics including cortical folding, sensory processing, and the contributions of various brain regions such as the cerebellum and brainstem. The role of mirror neurons and other neural systems in shaping social behavior is discussed, alongside insights from animal models that have advanced our understanding of autism’s underlying mechanisms. Ultimately, this manuscript argues that autism is not merely a biomedical challenge, but a broader societal issue intersecting with education, human rights, and identity. Following the long and winding road of scientific discovery, we advocate for a more empathetic, interdisciplinary, and human-centered approach to autism research. Though the path ahead remains uncertain, every step informed by evidence and driven by collaboration brings us closer to deeper understanding, greater inclusion, and more effective support.

## 1. Introduction

At first glance, the title of this manuscript might seem susceptible to plagiarism concerns, given its inspiration from The Beatles’ iconic song. However, this choice is intentional and serves a deeper purpose: it reflects the arduous, nonlinear journey of autism research, a journey as complex and multifaceted as Autism Spectrum Disorder (ASD) itself. ASD presents challenges across neurobiological, behavioral, and sociocultural dimensions, affecting individuals, families, and societies alike. Despite advances in characterizing its genetic underpinnings, sensory processing differences, and social communication profiles, the etiology of ASD remains elusive, and its heterogeneity defies simplistic explanations. This review navigates the long and winding road of ASD research, weaving together current findings, unresolved questions, and emerging frameworks that may guide future inquiry.

The metaphor of a journey, structured around Beatles song titles, mirrors the iterative, often circuitous nature of scientific progress in ASD, where breakthroughs are interwoven with setbacks and paradigm shifts. This narrative strategy is intentionally designed for a multidisciplinary readership, encompassing neuroscientists, clinicians, educators, policymakers, and families. It also acknowledges the cultural and emotional resonance of music, a universal language that parallels the urgent need for interdisciplinary dialogue in autism research. While this manuscript is rooted in empirical evidence, the deliberate use of metaphor aims to humanize the scientific narrative, bridging gaps between clinical rigor and the lived experiences of autistic individuals and their families. Each section explores a distinct “mile marker” on this road, underscoring both the progress made and the distance yet to travel. To chart this path, we begin with Yesterday, a retrospective analysis of autism’s historical discovery and evolving definitions, before turning to other sections, each analyzing a distinct waypoint on this road.

## 2. Yesterday

Paul Eugen Bleuler, a Swiss psychiatrist specializing in schizophrenia, published his landmark work Dementia Praecox or the Group of Schizophrenias in 1911 (as translated into English). This work laid the foundation for modern understandings of schizophrenia and its associated symptoms. A century later, Moskowitz and Heim [1] published a centennial analysis of Bleuler’s contributions, reaffirming that schizophrenia was indeed the central focus of his studies. In his foundational work, Bleuler introduced what he termed the “4 A’s”—affect, associations, ambivalence, and autism—which became key features in the characterization of schizophrenia. Notably, this marked the first recorded instance where the term “autism” was used. However, it did not refer to autism as we understand it today. Instead, Bleuler used the term to describe a withdrawal from reality, which he considered a secondary feature of schizophrenia.

Building on this, in 1926, Russian child psychiatrist Grunya E. Sukhareva advanced the understanding of what we now know as autism. In her publication Die schizoiden Psychopathien im Kindesalter (Schizoid Psychopathies in Childhood), Sukhareva described the behaviors of children exhibiting schizoid characteristics and introduced the term “autistic psychopathology”. Her work represented a significant shift, as she focused more on social and motor difficulties, core features that resonate with the contemporary definition of autism. Sukhareva’s observations emphasized the importance of these traits in the children she studied, effectively pioneering the understanding of autism as distinct from schizophrenia, even though the terminology at the time was still evolving [2]. Sukhareva’s early work, much like Bleuler’s, did not gain widespread recognition immediately, yet it laid important groundwork for later developments in the history of autism research.

Several years later, Leo Kanner published his groundbreaking work Autistic Disturbances of Affective Contact [3,4], where he studied a cohort of eleven children, aged between two and eight years, who exhibited similar patterns of behavior, leading to the identification of what would later be known as Kanner Syndrome, or early infantile autism. In his study, Kanner defined autism as a distinct neurodevelopmental disorder, characterized by an innate inability to form typical social connections. He emphasized that these children displayed a profound lack of interest in social interactions and emotional engagement with others, a hallmark trait that would shape the understanding of autism for decades. Additionally, Kanner identified key features of the condition, including restricted and repetitive behaviors, challenges in communication, and a tendency towards extreme aloneness. These characteristics remain central to the modern conceptualization of ASD. Kanner also noted the heterogeneity within the disorder, observing that while all the children shared core symptoms, there were significant variations in the severity and manifestation of these behaviors across individuals. Despite these differences, Kanner argued that these children shared fundamental commonalities from a psychopathological perspective. Importantly, he advocated for a cautious approach to diagnosis, stressing the necessity of treating each child as an individual, rather than rigidly categorizing them within a fixed diagnostic framework. His emphasis on the complexity and variability of autism underscored the need for a nuanced, personalized approach to each case. Kanner’s work laid the foundation for the formal recognition of autism in psychiatric classifications, which culminated in the inclusion of infantile autism in the DSM-III in 1980, solidifying autism’s place as a recognized clinical entity.

In 1944, pediatrician Hans Asperger identified a group of children exhibiting distinctive behaviors such as difficulties in social interactions, a lack of empathy, and an intense focus on specific interests. However, his observations did not gain formal recognition until Asperger Syndrome (AS) was included in the Diagnostic and Statistical Manual of Mental Disorders, Fourth Edition (DSM-IV) in 1994. This inclusion marked a significant milestone by officially categorizing AS as a distinct diagnosis within the broader spectrum of pervasive developmental disorders. The recognition of AS in the DSM-IV led to increased awareness and research, significantly expanding the literature and understanding of the syndrome. This surge in interest prompted numerous publications discussing AS’s implications for education, therapy, and support [5,6,7]. Despite this progress, controversy arose regarding the overlap between AS and high-functioning autism, sparking debates about the validity of AS as a separate diagnosis. Critics argued that the distinctions between AS and autism were often unclear, as both groups exhibited similar social and behavioral challenges, raising questions about AS’s independent existence. To address these ambiguities and unify various autism-related diagnoses, AS was subsumed under the broader category of ASD in the fifth edition of the DSM (DSM-5) in 2013 [8,9]. This change reflected a growing recognition that autism encompasses a spectrum of experiences with varying degrees of severity and characteristics. Consequently, the formal recognition of AS as a separate diagnosis lasted approximately 19 years, from 1994 to 2013, before being integrated into the more inclusive framework of ASD.

Now a decade old, the DSM-5 (2013) remains the primary framework for diagnosing ASD, which is characterized by a spectrum of behavioral manifestations that typically emerge in early childhood and diverge from typical developmental patterns. The diagnostic criteria are organized into two primary domains: deficits in social communication and interaction, and restricted and repetitive behaviors and interests. The first domain focuses on challenges in social communication and interaction, where individuals with autism may find it difficult to initiate or respond to social exchanges. These challenges often encompass both verbal and non-verbal communication, including limited use of gestures, facial expressions, and body language. Individuals may struggle to make eye contact, interpret social cues, or maintain appropriate responses in various social contexts. Additionally, understanding or expressing emotions can pose significant challenges, complicating reciprocal interactions. Many individuals with ASD also exhibit limited imaginative play and show reduced interest in forming relationships, which can vary in severity and often lead to substantial difficulties navigating social environments. The second domain includes restricted and repetitive behaviors and interests. These behaviors can manifest in several ways, such as echolalia (the repetition of words or phrases), repetitive movements (e.g., hand-flapping), and strict adherence to routines, which can result in inflexibility when adapting to changes in daily life. Individuals may develop intense, focused interests in specific topics or objects, often to the exclusion of other activities. Sensory perception is also a common feature of autism; individuals may exhibit heightened or diminished responses to stimuli such as sound, temperature, or pain. A fascination with visual stimuli, like lights or patterns, is frequently observed.

On the other hand, the DSM-5 also outlines three levels of support for individuals with ASD, which help determine the amount of assistance required across the two main domains. These levels provide a framework for understanding the degree of support needed to help individuals manage their challenges and live as independently as possible.

Level 1: Requiring Support. At this level, individuals may face social challenges but can function relatively independently with some assistance. They might need support to initiate conversations, respond appropriately in social interactions, and sustain engagement in conversations. Behavioral patterns at this level tend to be rigid, and individuals may struggle to cope with changes in routine or unexpected situations. They also may need help organizing and planning daily activities. Overall, individuals at this level require minimal but consistent support to navigate social and behavioral challenges.

Level 2: Requiring Substantial Support. Individuals at Level 2 need more assistance than those at Level 1, particularly in social situations. Holding conversations can be very difficult, even with support, and speech may be limited to short sentences or very specific topics. Nonverbal communication, such as understanding facial expressions or gestures, is often challenging. Individuals at this level may experience significant distress when faced with changes in routine or environment, and their repetitive behaviors can interfere with daily functioning. More structured and consistent support is needed to help them navigate both social and behavioral difficulties.

Level 3: Requiring Very Substantial Support. This represents the highest level of need, where individuals require the most significant support. Verbal and nonverbal communication are severely impaired, making it difficult to engage in or understand social interactions. These individuals may avoid or limit interactions with others, show little interest in forming friendships, and find it extremely difficult to engage in imaginative play. They also exhibit highly restrictive and repetitive behaviors that profoundly impact their ability to function in daily life. Changes to routine can cause extreme distress, further underscoring the need for substantial, ongoing support.

It is important to note that the level of support required can vary across the two main domains. For instance, an individual might require Level 1 support in social communication but need Level 2 support for managing restricted and repetitive behaviors. This variability highlights the importance of tailoring support to the specific needs of each individual, ensuring that the right level of assistance is provided to help them manage their challenges, develop skills, and achieve the highest degree of independence possible.

This brief exploration of the history of ASD is essential for understanding the wide range of manifestations that characterize the spectrum, highlighting the diverse social and behavioral challenges faced by individuals with autism. The variability in how autism presents means that each person experiences the condition uniquely, with distinct symptoms and varying support needs. Consequently, autism requires a flexible, individualized approach to intervention and therapy tailored to address each person’s specific requirements. Understanding these complexities is vital for developing strategies that not only enhance the quality of life for individuals on the spectrum but also promote an inclusive environment that acknowledges their strengths and effectively addresses their unique challenges.

## 3. Dig It

The etiology of ASD can be categorized into three distinct subgroups—symptomatic, cryptogenic, and idiopathic—based on the presence or absence of identifiable underlying causes [10]. Symptomatic ASD encompasses cases where autism symptoms are associated with clear neuro/medical conditions or diagnoses, such as neurodevelopmental disorders, genetic syndromes, or psychiatric comorbidities. Cryptogenic ASD refers to cases where underlying causes are suspected but remain unidentified due to a lack of definitive evidence. In contrast, idiopathic ASD applies to children for whom no identifiable cause can be determined. These classifications are essential for understanding the complex heterogeneity of ASD. Beyond this classification, the etiology of ASD is inherently complex and multifactorial, involving a delicate interplay of genetic, environmental, and neurodevelopmental factors. Genetic components play a pivotal role, with numerous studies identifying a wide range of genes associated with increased autism risk. These include genes involved in neurodevelopment, synaptic function, and neurotransmitter systems [11,12,13]. Environmental influences, particularly during prenatal and early postnatal development, also significantly contribute to ASD risk. Key environmental factors include maternal infections during pregnancy, exposure to teratogens or certain medications, advanced parental age, and prenatal stress such as maternal immune activation [14]. The intricate interactions between genetic and environmental factors highlight the multifaceted origins of ASD. Disruptions during early neurodevelopment often lead to atypical brain development and the diverse behavioral manifestations of the disorder [15]. Given the variability in triggers and contributing factors, the subsequent Dig It sections will delve into the genetic and environmental effects on the neurodevelopmental aspects of ASD.

### 3.1. Genes

**Figure 1:**
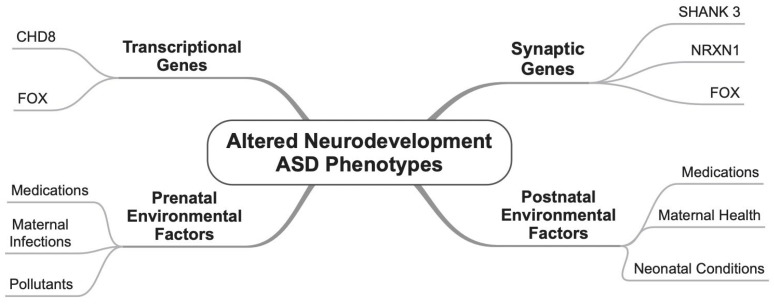
Gene-environment interactions in Autism Spectrum Disorder (ASD). This schematic highlights the interplay between genetic factors (synaptic and transcriptional genes), and prenatal or postnatal environmental influences, which together disrupts multiple neurodevelopmental processes and contribute to the emergence of ASD phenotypes.

Although databases such as SFARI Gene (https://www.sfari.org/resource/sfari-gene/ (accessed on 2 September 2025)) and AutDb (http://autism.mindspec.org/autdb/Welcome.do (accessed on 2 September 2025)) report over 1400 genes with a potential role in the underlying mechanisms of autism, a growing body of research has focused on 215 high-confidence risk genes associated with ASD. These genes underscore the significant genetic heterogeneity of the condition. High-confidence risk genes are validated through rigorous evidence, including genetic studies, functional analyses, and replication across diverse populations and datasets. Among these, CHD8, SHANK3, NRXN1, and the FOXP family, have consistently been strongly linked to ASD in numerous studies. Additionally, genes such as WAC, GRIN2B, and DYRK1A have emerged as critical contributors to the genetic architecture of autism, often showing substantial overlap across various research efforts. This genetic complexity highlights the importance of continued investigation into these risk genes to deepen our understanding of their roles in the etiology of ASD. Brief information about some of these genes is provided next (Figure 1).

CHD8 (Chromodomain Helicase DNA Binding Protein 8) is a member of the CHD protein family and functions as an ATP-dependent DNA helicase involved in chromatin remodeling. This protein plays a crucial role in regulating gene expression and cellular proliferation during neurodevelopment. The CHD8 gene is located on chromosome 14 at the 14q11.2 region [16] and is the most frequently mutated gene in autism and other neurodevelopmental disorders [17,18]. Mutations in CHD8 have been strongly associated with a subset of autism cases, often presenting with macrocephaly, developmental delay, ataxia, and distinct facial features. These genetic alterations are also linked to comorbid conditions, such as attention-deficit hyperactivity disorder (ADHD) and cardiovascular abnormalities [19]. The central role of CHD8 in neurodevelopment highlights its importance in understanding the genetic basis of autism.

SHANK3 (SH3 and Multiple Ankyrin Repeat Domains 3) is a gene that encodes a synaptic scaffolding protein, also called SHANK3, located on chromosome 22 in the 22q13.3 region [20]. This protein is essential for the development, maturation, and maintenance of excitatory synapses in the brain. As a major component of the postsynaptic density (PSD), SHANK3 interacts with a wide array of other synaptic proteins to organize and stabilize the molecular architecture of dendritic spines. The PSD is a highly specialized subcellular structure enriched with proteins involved in synaptic signaling, receptor trafficking, and cytoskeletal dynamics, all of which are critical for synaptic function and plasticity [21]. Mutations and deletions in the SHANK3 gene have been linked to several neurodevelopmental disorders. One of the most prominent is 22q13.3 deletion syndrome, commonly known as Phelan-McDermid syndrome. This rare genetic condition is characterized by severe expressive language delays, global developmental delays, intellectual disability, and hypotonia [22]. Individuals with this syndrome often exhibit ASD-like behaviors, including deficits in social communication and restricted or repetitive interests. The link between SHANK3 disruptions and these phenotypes highlights the gene’s role in neuronal connectivity and communication. Moreover, mutations in the SHANK3 gene have been identified in both humans and animal models with ASD, further underscoring its significance in neurodevelopment [23].

NRXN1 (Neurexin 1) is a gene that plays a role in the genetics of ASD and other neurodevelopmental conditions. This gene encodes a presynaptic adhesion protein critical for neurotransmitter release and synaptic organization. Mutations, deletions, or disruptions in NRXN1 at the 2p16.3 region of chromosome 2 have been strongly linked to ASD, intellectual disabilities, and schizophrenia [24,25]. Although NRXN1 mutations are considered high-confidence risk factors for ASD, their prevalence is relatively low, found in less than 7% of autistic individuals [26]. The rarity of NRXN1 mutations highlights the importance of considering genetic heterogeneity in ASD. Studies using animal models, particularly mice, have provided valuable insights into the consequences of NRXN1 alterations. Mice with reduced or absent NRXN1 expression often exhibit a range of behaviors resembling core symptoms of autism, including impaired social interactions, communication deficits, and repetitive behaviors [24,27,28]. These phenotypes can vary depending on the specific nature of the genetic alteration, such as whether it involves a partial deletion, a point mutation, or a complete knockout. Intriguingly, sex differences have been observed in some studies, suggesting that male and female individuals might be differentially affected by NRXN1 disruptions [29,30]. Beyond its specific link to ASD, the role of NRXN1 in synaptic function suggests broader implications for understanding the neurobiological mechanisms underlying various neurodevelopmental and psychiatric disorders. Its interaction with other synaptic proteins, such as neuroligins and SHANK proteins [31], further emphasizes the interconnected nature of synaptic networks and their vulnerability to genetic perturbations.

The FOX (Forkhead box) gene family is an evolutionarily ancient group of genes present across various species, from yeast to humans, and is divided into subfamilies such as FOXA, FOXO, and FOXP [32]. The FOXP subfamily includes FOXP1, FOXP2, FOXP3, and FOXP4, located at 3p14.1, 7q31.1, Xp11.23, and 6p25.3, respectively [32,33]. Among these, FOXP1, FOXP2, and FOXP4 are of particular interest due to their roles in development and cognition. Dysregulation or mutations in these genes have been linked to ASD and related neurodevelopmental conditions [34]. FOXP1 is expressed in brain regions such as the cortex, striatum, and hippocampus, where it regulates neuronal signaling, synapse formation, and cognitive function; its mutations or deletions are associated with language deficits, impaired social behavior, and developmental delay, all characteristic of ASD [35]. FOXP2 is best known for its role in speech and language acquisition, with mutations linked to developmental verbal dyspraxia, a condition involving difficulties in coordinating motor movements required for speech [36]. Functional studies show FOXP2 regulates genes crucial for synaptic plasticity, implicating its role in the communication deficits seen in ASD [37]. FOXP4, the least studied member of the FOXP family, is expressed in the brain and shares overlapping expression patterns with FOXP1 and FOXP2. Although its specific role remains unclear, its identification in some ASD cohorts suggests a potential contribution to the disorder, warranting further investigation [34,38].

The examples provided offer a glimpse into the roles of specific genes in ASD. However, the field of autism genetics is vast and rapidly evolving, with ongoing research continually uncovering new associations and mechanisms. A deeper understanding of these genes and their interactions is critical for unraveling the molecular and cellular complexities underlying ASD. This growing knowledge underscores the intricate nature of the condition and highlights the importance of further studies to fully elucidate the genetics of autism.

### 3.2. Environmental Factors

The prenatal environment is a critical determinant of fetal neurodevelopment, influencing the intricate processes that shape the developing brain. Numerous prenatal factors have been associated with an increased risk of ASD, often interacting with genetic predispositions [39] to amplify the likelihood of atypical neurodevelopment. Among these factors, maternal obesity has emerged as a significant contributor to ASD risk. Studies report that children born to obese mothers exhibit a 47% higher likelihood of developing ASD compared to those born to mothers of normal weight [40]. In addition to obesity, other metabolic conditions, such as hypertension and diabetes, have also been linked to an increased risk of ASD [41]. These conditions may create a suboptimal intrauterine environment, characterized by inflammation, oxidative stress, and altered nutrient supply, which can adversely impact fetal brain development. Maternal infections during pregnancy, particularly those occurring in the first and second trimesters, are another critical factor associated with ASD risk [42]. Viral infections, in particular, can trigger maternal immune activation, leading to elevated levels of pro-inflammatory cytokines that cross the placental barrier. These cytokines may disrupt normal brain development, potentially contributing to the emergence of autistic traits [43]. Moreover, the timing and severity of these prenatal exposures can influence the extent of neurodevelopmental disruptions. For instance, studies have shown that early gestational infections may impact neural tube formation, while infections later in pregnancy might affect synaptogenesis and cortical organization [44].

Prenatal exposure to environmental toxins has emerged as a critical area of research in understanding ASD, with studies highlighting the potential risks posed by specific pollutants. Among these, traffic-related air pollution (TRP) has garnered significant attention. Evidence suggests that children exposed to the highest levels of TRP during prenatal development or their first year of life are more likely to develop ASD compared to those with lower exposure levels [45]. This research underscores the role of air quality in autism risk, identifying associations between ASD and key pollutants such as nitrogen dioxide, fine particulate matter, and coarse particulate matter. These fine particles, capable of crossing the placental barrier, may disrupt fetal neurodevelopment, thereby increasing the risk of autism.

Pesticides are also considered plausible contributors to ASD. Animal studies point to critical windows of exposure during development, though the specific periods of susceptibility in humans remain unclear. Pesticide exposure frequently occurs in combination with other environmental chemicals, such as flame retardants and plasticizers, complicating the identification of their individual and combined effects on autism risk [46]. Epidemiological studies have linked gestational exposure to certain pesticides with an increased likelihood of ASD diagnoses in children [47]. Specific pesticides, including glyphosate and chlorpyrifos, have been associated with heightened ASD risk, particularly during critical periods of neurodevelopment. Additionally, pyrethroids have been linked to behavioral and executive functioning deficits commonly observed in individuals with ASD [48].

Heavy metal exposure is another significant area of concern in ASD research [49]. Among heavy metals, lead is prominently implicated, with evidence suggesting that even low-level exposure can negatively affect cognitive function. Methylmercury is recognized for its neurodevelopmental risks, particularly during prenatal and early childhood exposure. Cadmium, while less extensively studied, is acknowledged for its potential to cause neurodevelopmental damage. Similarly, manganese exposure has been associated with hyperactivity and cognitive impairments in children, further emphasizing the impact of heavy metals on neurodevelopment.

Certain prenatal medications have been linked to an increased risk of ASD in children. One prominent example is valproate, an anticonvulsant, which is strongly associated with a significantly heightened risk of ASD. Research indicates that children exposed to valproate in utero are substantially more likely to develop ASD compared to those not exposed [50]. On the other hand, the use of selective serotonin reuptake inhibitors (SSRIs) during pregnancy has produced mixed results regarding autism risk. Some studies suggest a modest increase in risk, particularly when SSRIs are used during the first trimester, a critical period for neurodevelopment [51]. However, the evidence remains inconclusive, warranting further investigation.

Beyond medications, maternal nutrition plays a pivotal role in fetal brain development and may influence ASD risk. A deficiency in folic acid during early pregnancy has been linked to a higher likelihood of ASD, with research showing that mothers who do not take prenatal vitamins containing folic acid are at increased risk of having a child with autism [52]. Similarly, vitamin D deficiency during pregnancy has been associated with autism-like traits in offspring. Studies have found that children born to vitamin D-deficient mothers tend to score higher on autism-related behavioral measures [53].

Postnatal influences on ASD have also garnered increasing attention, with research identifying various factors that may contribute to the risk of developing this condition. Key postnatal factors associated with autism include low birth weight, postpartum complications, and specific neonatal conditions. For example, children born with low birth weight have been shown to have a significantly higher risk of ASD, with studies indicating correlations between low birth weight, postpartum hemorrhage, and other neurodevelopmental challenges [54]. Additionally, postnatal exposures such as certain medications, including acetaminophen and antibiotics, as well as conditions like ear infections or decreased breastfeeding, have been linked to an elevated risk of autism [55]. Emerging evidence also highlights the potential impact of maternal health during the postpartum period on a child’s neurodevelopment. Maternal postpartum depression, in particular, has been associated with an increased risk of ASD in offspring. A study revealed that children raised by mothers experiencing postpartum depressive symptoms had a heightened likelihood of developing autism [56].

In brief, the intricate interplay between prenatal and postnatal factors underscores their pivotal roles in shaping ASD risk, driven by a combination of genetic susceptibility and environmental influences. Prenatal exposures to harmful toxins, such as air pollution, pesticides, and heavy metals, along with maternal health and behaviors, establish the foundation for neurodevelopment, where disruptions during these sensitive periods can have profound and lasting effects. Postnatal factors, including low birth weight, neonatal complications, and maternal postpartum health, further highlight the vulnerability of early developmental windows (Figure 1). These findings emphasize the importance of adopting a holistic, life-course approach to understanding ASD, with a focus on implementing preventive strategies and targeted interventions from conception through early childhood to promote optimal neurodevelopment.

## 4. Boys

Autism exhibits a notable gender disparity, with boys being diagnosed significantly more often than girls. Across studies, the male-to-female ratio varies widely, ranging from 0.8:1 to as high as 6:1 [57], though it is most commonly reported as 4:1 or 3:1 [58], depending on the population and diagnostic methods employed. This imbalance has sparked extensive investigation into the underlying biological and genetic factors that may drive the disparity. One key area of focus is the role of sex chromosomes. Research suggests that males may be biologically predisposed to neurodevelopmental disorders like autism due to their chromosomal makeup. The pronounced male excess is thought to be influenced by genetic factors linked to the X chromosome, which may increase vulnerability to ASD in males. Indeed, studies have identified significant associations between specific X chromosome variants and autism [59]. Further insights come from research analyzing the disparity between the sex ratio at conception (SRC) and the sex ratio at birth (SRB). While the SRC is approximately equal for male and female embryos, the SRB skews slightly toward more male births, suggesting that biological factors may influence the survival of male embryos during early developmental stages [60]. Male embryos may have a selective advantage due to faster cell proliferation rates, a trait potentially linked to the presence of the SRY gene on the Y chromosome, which determines male sex. Although fewer studies have explored the role of the Y chromosome in ASD risk, emerging evidence highlights its potential significance. For example, individuals with an additional Y chromosome are more likely to receive an ASD diagnosis compared to those with a typical chromosomal pattern [61,62]. This finding challenges the assumption that the Y chromosome is protective and suggests it may harbor risk factors that contribute to ASD.

Hormones, particularly sex hormones like testosterone, are hypothesized to play a pivotal role in the male predominance of ASD. One influential framework, the Extreme Male Brain (EMB) theory, posits that ASD reflects an amplification of typical male cognitive traits, characterized by heightened systemizing—the drive to analyze or construct systems—and reduced empathizing, or the ability to recognize and respond to others’ emotions [63,64]. This theory suggests that elevated prenatal testosterone exposure may shape these cognitive patterns, potentially contributing to the higher prevalence of ASD in males [65]. While the EMB theory has garnered empirical support, it remains a subject of debate, yet it continues to influence how researchers conceptualize the cognitive and behavioral characteristics associated with autism [66,67]. Beyond cognition, prenatal testosterone has been implicated in structural and functional alterations in brain regions critical to ASD-related behaviors. Elevated testosterone levels are associated with atypical development of the amygdala, a key hub for social and emotional processing [68,69]. Additionally, prenatal testosterone may dysregulate the hypothalamic-pituitary-adrenal (HPA) axis, potentially contributing to heightened anxiety and social challenges in ASD [70].

Testosterone alone does not fully account for the sex bias in ASD. Estrogen, traditionally viewed as a female hormone, plays a neuroprotective role in brain regions implicated in ASD, such as the prefrontal cortex (critical for executive function) and the amygdala (central to emotional processing). Preclinical studies show that estrogen increases parvalbumin—a calcium-binding protein—ameliorating ASD-like behaviors, including social deficits and repetitive grooming, in rodent models [71]. Dysregulation of estrogen receptors (ERα and ERβ) has also been linked to ASD symptoms, particularly impaired synaptic plasticity and social communication. Estrogen further enhances social behavior by upregulating oxytocin receptor expression in key brain regions such as the hypothalamus and nucleus accumbens, thereby strengthening circuits essential for social affiliation [72,73]. Oxytocin, a hormone central to social bonding, is strongly implicated in ASD pathophysiology. Males with ASD frequently exhibit lower baseline plasma oxytocin levels compared to neurotypical peers, and these deficits correlate with reduced social reciprocity and impaired emotional recognition [74]. However, oxytocin’s effects are highly context-dependent, and clinical trials of intranasal oxytocin have produced inconsistent therapeutic outcomes [75]. Beyond hormonal influences, prenatal environmental factors also contribute to ASD’s male bias. Variations in maternal cortisol levels during pregnancy—a marker of maternal stress—have been associated with an increased risk of ASD in male offspring [76,77]. Additionally, maternal hypothyroidism during pregnancy has been identified as another risk factor, with hypothyroxinemia linked to a higher likelihood of ASD in children [78].

Sex-specific neurological variations are crucial in shaping the differential manifestation of ASD. One notable difference is observed in the amygdala, which shows accelerated growth in males with ASD. This enlargement may heighten neural sensitivity to social stimuli and potentially intensify core symptoms [68]. Similarly, the prefrontal cortex (PFC) exhibits sex-specific modifications. Males with ASD typically present with distinct cytoarchitectural changes in the PFC, including alterations in cortical thickness and increased neuronal soma size. These changes may contribute to difficulties in processing social cues and engaging in effective social interactions [79]. Interhemispheric connectivity further highlights these differences. In males with ASD, reductions in corpus callosum size have been linked to impairments in social behavior, communication, and cognitive processing [80]. Network connectivity patterns offer additional insights. Males frequently display hyperconnectivity within local neural circuits coupled with underconnectivity in long-range circuits, a combination that can disrupt attention and social functioning [81]. Finally, while the salience network—which is critical for detecting and prioritizing relevant stimuli—is altered in ASD, these changes appear to be similar across sexes. In autism, the salience network tends to show increased connectivity with sensorimotor regions and reduced connectivity with prefrontal areas [82].

## 5. Girl

Building on prior discussions, emerging research increasingly challenges the longstanding belief that autism is significantly less common in females. While historical estimates have reinforced this notion, recent evidence suggests that diagnostic biases and the subtler presentation of autistic traits in girls may contribute to these skewed statistics. The Female Protective Effect (FPE) proposes that females have a higher genetic threshold for developing ASD than males, meaning they require a greater accumulation of genetic risk factors before exhibiting traits that meet clinical diagnostic criteria [83]. As a result, some females may carry autism-associated genetic variants but remain undiagnosed due to milder or atypical manifestations. Supporting this hypothesis, genes downregulated in ASD post-mortem brains, many involved in synaptic function and neuronal communication, tend to be more highly expressed in female brains [84]. This suggests that females may have a stronger baseline expression of genes that support neural connectivity, potentially buffering them from the full clinical presentation of ASD despite the presence of underlying risk factors.

Beyond these biological dimensions, girls with autism often exhibit subtler or atypical traits, such as less overt repetitive behaviors and socially acceptable special interests. They also frequently engage in “masking” or “camouflaging”—conscious or unconscious strategies used to conceal or compensate for autistic traits in social situations. This includes mimicking neurotypical behaviors, suppressing stimming, and using rehearsed social scripts to navigate interactions [85]. Research suggests that females with ASD camouflage more frequently than males, contributing to underdiagnosis or delayed diagnosis, as their symptoms often go unnoticed or are misattributed to other conditions [86]. While camouflaging can facilitate social integration, it often comes at a significant emotional cost, leading to mental health distress that compromises daily functioning [87].

Diagnostic procedures, including the widely used second edition of the Autism Diagnostic Observation Schedule (ADOS-2), may contribute to discrepancies in autism diagnoses between males and females due to differences in how autistic traits manifest across sexes. Autistic females often exhibit fewer or less pronounced behaviors characteristic of ASD, are more likely to camouflage their symptoms, employ compensatory social strategies, and display interests that align with neurotypical peers. These factors can make their autistic traits less detectable by standard diagnostic measures like the ADOS-2 [88]. Moreover, since the ADOS-2 was primarily developed based on male presentations of autism, it may be less sensitive to the social and behavioral characteristics of autistic females [89]. This diagnostic bias can lead to underdiagnosis or delayed diagnosis in females, limiting their access to early interventions and support services. Consequently, while historical estimates suggest a higher male-to-female ratio, revised epidemiological approaches and improved diagnostic sensitivity increasingly indicate that prevalence rates between sexes may be more comparable than previously thought.

## 6. What Goes on

For decades, researchers have probed the neurological foundations of autism, seeking to understand how brain structure and connectivity differ in autistic individuals. Advances in neuroimaging and developmental neuroscience have illuminated key features, yet the emerging picture remains both complex and multifaceted. Autism is increasingly recognized as involving distinct patterns of neural organization that shape cognition, sensory processing, and social interaction in ways that diverge from typical development. Research has identified neuroanatomical variations such as early childhood brain overgrowth, altered synaptic pruning, and differences in white matter organization. Neuroimaging studies further reveal that while some individuals exhibit increased cortical thickness or atypical volumes in subcortical regions, others demonstrate unique connectivity patterns suggestive of divergent neural network development. These diverse findings underscore the remarkable heterogeneity of the autism spectrum; for example, while some children display transient brain enlargement, others show lifelong differences in regional communication—evidence that autism’s neurobiology defies a single explanatory narrative (Table 1). This section synthesizes current research on the structural and connectivity-related changes in autistic brains.

### 6.1. Head Circumference

Research into head circumference (HC) growth in children with ASD indicates that approximately 14–34% of affected children develop macrocephaly. Although HC is typically normal at birth, children who later exhibit macrocephaly (ASD-M) experience accelerated head growth within the first six months—a trend that continues throughout early childhood. Intriguingly, these children have been observed to demonstrate better language, social communication, and emotional skills, suggesting that early brain overgrowth does not necessarily correlate with more severe ASD symptoms [90]. Magnetic resonance imaging studies further reveal that by ages 2–4 years, autistic toddlers have brain volumes approximately 10% larger than those of their typically developing peers, with particularly pronounced enlargement in the cerebrum, cerebellum, and limbic structures—especially within the frontal and temporal lobes. However, following this initial surge, brain growth slows markedly. This abrupt deceleration likely disrupts neural circuit formation, potentially contributing to the atypical behaviors associated with autism [91]. Notably, while macrocephaly is a frequent finding, a subset of children with ASD exhibit microcephaly. Unlike macrocephaly, microcephaly is strongly linked to co-occurring medical disorders and intellectual disability, with all microcephalic children showing significant cognitive impairments. Although there is a slight trend toward higher rates of microcephaly among females and lower-functioning individuals, these associations did not reach statistical significance [92]. The coexistence of both macrocephaly and microcephaly in autism suggests that variations in head circumference reflect different underlying neurobiological pathways, potentially driven by distinct genetic or environmental influences on brain development.

### 6.2. Cerebral Cortex

The cerebral cortex, an intricately folded sheet of neural tissue, represents the pinnacle of anatomical and functional complexity within the central nervous system [93]. This outermost brain layer orchestrates higher-order cognitive processes, including perception, attention, memory, language, and consciousness. Its multilaminar architecture—organized into six histologically distinct layers comprising specialized neuronal populations and synaptic networks [94]—enables both hierarchical and parallel information processing essential for adaptive behavior. Anatomically, the cortex is subdivided into four primary lobes—frontal, parietal, temporal, and occipital—based on sulcal landmarks and functional specialization. Additionally, the insular cortex, located deep within the lateral sulcus, is often considered a fifth lobe due to its distinct cytoarchitecture and unique functional contributions [95]. Each lobe contributes differentially to cognition, and their integrated activity underlies coherent and flexible mental functioning.

In individuals with ASD, converging evidence from neuroimaging and postmortem studies reveals widespread deviations in cortical development, including abnormal folding patterns (gyrification), cortical thickness variations, and altered connectivity [96,97]. Gyrification, which increases cortical surface area within the limited intracranial volume, is frequently atypical in ASD—particularly in the frontal and temporal regions that subserve executive functions and social cognition [98,99]. For instance, aberrant folding in the frontal cortex may impair the maturation of neural circuits involved in decision-making and social behavior, whereas anomalies in the temporal lobe are linked to deficits in language acquisition and affective processing [100,101]. These macrostructural alterations are thought to arise from multifactorial etiologies: genetic susceptibilities—such as mutations in genes like *SHANK3*, *NLGN3*, or *CNTNAP2* that regulate synaptogenesis—interact with prenatal environmental insults, including maternal immune activation or toxic exposures, thereby perturbing critical neurodevelopmental pathways [102].

At the microstructural level, the cerebral cortex is organized into vertically oriented minicolumns—repetitive, modular units that integrate local and long-range synaptic inputs. ASD is associated with atypical minicolumnar organization, though findings vary across studies. Initial investigations reported narrower minicolumns with dispersed cells, suggestive of imbalanced connectivity patterns [103]. In contrast, later studies observed wider minicolumns in younger individuals with ASD, representing less overlap of dendritic trees and in turn less co-activation of neighbor neurons [104]. Cortical thickness also exhibits regional heterogeneity, as individuals with autism are often altered, reflecting developmental abnormalities that may impact cognitive and behavioral functions [105]. These microanatomical anomalies likely arise from disrupted processes such as neuronal migration [106], programmed cell death [107], and glial-mediated circuit refinement during early brain development [108]. Functional neuroimaging studies further demonstrate aberrant patterns of cortical connectivity in ASD, encompassing both hypo- and hyperconnectivity across brain networks [109].

### 6.3. Basal Ganglia

The basal ganglia comprise several interconnected subcortical nuclei, the striatum (caudate nucleus and putamen), nucleus accumbens, globus pallidus, subthalamic nucleus, and substantia nigra, that support motor control, higher-order cognition, social interactions, speech, and repetitive behaviors [110]. In ASD, structural and functional alterations have been documented across these nuclei. Volumetric changes are most consistently observed in the caudate nucleus: some studies report increased caudate volume, while others see no difference or enlargement proportional to overall brain size [111,112]. Caudate volume correlates significantly with the severity of ritualistic-repetitive behaviors [111,113]. Longitudinal research even indicates that changes in the caudate nucleus development during early childhood may be implicated in the emergence of restricted and repetitive behaviors [114]. Gray matter increases have also been found in the putamen and thalamus [111,115].

Functionally, atypical frontostriatal activation is evident during cognitive-control tasks related to restrictive and repetitive interests [116]. Furthermore, reduced connectivity between the caudate nucleus and prefrontal cortex may contribute to cognitive inflexibility in ASD. Conversely, some studies examining motor tasks have demonstrated striatal hyperactivity, suggesting an overactive basal ganglia [111]. At a neurochemical level, a loss of the GABA-synthesizing enzyme GAD67 in striatal neurons and cerebellum has been linked to social deficits, hyperactivity, and cognitive rigidity [110].

### 6.4. Amygdala

The amygdala has long been considered a key brain area implicated in ASD. An influential theory, coined the Amygdala Theory of Autism, posits that the social dysfunctions observed in ASD could be caused by an abnormality in the amygdala. This idea is based on the fact that childhood autism involves deficits in social intelligence, and the amygdala is part of the network of neural regions that make up the social brain [117,118]. Consistent research demonstrates an accelerated volumetric growth phase in the amygdala during early childhood in individuals with autism, typically declining by middle childhood. This finding is corroborated by in vivo MRI studies and postmortem analyses, which reveal elevated neuronal counts in autistic children and adolescents but not adults [119]. Investigations employing functional neuroimaging studies have revealed that subjects with ASD exhibit substantially diminished amygdala activation during the processing of faces or engagement in social tasks, thereby shedding light on the functional role of the amygdala in ASD [120].

In addition to the aforementioned structural and functional alterations, certain sources propose an endocrine component involving the amygdala in ASD. This region contains neuropeptides, notably oxytocin, which is associated with social recognition and found to be diminished in autistic individuals. Moreover, the amygdala plays a critical role in the integration of internal physiological states with social environmental input, and its functional dysregulation is a robust finding in this population [121].

### 6.5. Hippocampus

The hippocampus is a critical brain region involved in episodic memory, spatial reasoning, pattern separation, and social interaction. Research increasingly suggests that its contribution to social interaction may be linked to behaviors altered in ASD. Consequently, the hippocampus has become a focal point in ASD research, with many studies indicating that structural and functional abnormalities in this region play a role in the development of the disorder [122,123].

Observed structural anomalies in ASD include inconsistencies in hippocampal volume. Investigations have yielded variable results, with some reporting increased hippocampal volume in children and adolescents with ASD, others demonstrating decreased volume, and still others indicating no significant volumetric differences relative to typically developing children [124,125]. Research examining left-right asymmetry in hippocampal volume development has also generated disparate findings, with some studies indicating larger right hippocampal volume in autistic children and others reporting an enlarged left hippocampus [126,127]. Magnetic resonance imaging has revealed a distinct anatomical abnormality in the dentate gyrus region of autistic patients. A postmortem study has documented hippocampal neuropathology in subjects with ASD, noting abnormally small and densely packed cells in the subiculum and CA1 regions [128]. ASD is associated with several structural changes in the hippocampus, but more research is needed to fully understand these modifications [129].

Functional modifications have been documented in individuals with ASD [130]. Investigations employing resting-state functional MRI have identified deviations in functional connectivity within neural networks implicated in language processing among infants exhibiting a heightened risk for ASD. Specifically, these deviations are characterized by diminished intrahemispheric connectivity between the auditory cortex and the hippocampus. Conversely, infants with a lower risk profile displayed heightened sensitivity to vocalizations within the right fusiform gyrus and left hippocampus [131]. Consequently, dysfunction in hippocampal network connectivity, as assessed by the temporal synchronization of activity between the hippocampus and other cerebral regions, has emerged as a pivotal domain of inquiry in the study of the autistic human hippocampus.

### 6.6. Thalamus

Traditionally known as a relay center, the thalamus has extensive connections with various cortical and subcortical regions, indicating its possible role in neural circuits relevant to ASD. In addition to its well-known function of relaying information, the thalamus is also involved in cognitive processes [132], potentially explaining some behavioral alterations observed in autism. No significant volumetric alterations of the thalamus in ASD have been documented in isolation. However, when thalamic volume is correlated with total brain volume, findings suggest atypical neural connectivity patterns [133,134].

Functional connectivity remains a primary focus of discussion, particularly concerning distinctions in thalamic connectivity within males diagnosed with ASD. These males exhibited diminished and less age-dependent increases in thalamic local connectivity, which correlated with the severity of autism symptoms. Notably, both male and female individuals with ASD displayed heightened connectivity between the thalamus and sensorimotor regions, including auditory, somatosensory, and interoceptive cortices, a connectivity that decreased with age in both cohorts. These observations challenge the established overconnectivity hypothesis in ASD, instead revealing a pattern characterized by local underconnectivity in the anterior thalamus alongside increased long-range connectivity with areas associated with sensory processing. The results imply that compromised thalamocortical communication may contribute to the perceptual and motor deficits observed in ASD, reflecting developmental delays in brain maturation [135].

ASD is associated with both anatomical and functional underconnectivity between the thalamus and various cortical areas. However, the temporal lobe, particularly the right side, shows increased functional connectivity. Microstructural studies point to impairment within the thalamic motor region, which may contribute to the motor challenges often seen in ASD. These connectivity patterns suggest that disruptions in thalamocortical pathways may be fundamental to the sociocommunicative, cognitive, and sensorimotor impairments characteristic of ASD. The strong connectivity between the temporal lobe and thalamus indicates a complex role for these pathways in the development and expression of the disorder [97].

### 6.7. Hypothalamus

Anatomical analyses indicate that individuals diagnosed with ASD exhibit hypothalamic atrophy, characterized by decreased gray matter volume and reduced neuronal density, particularly within nuclei implicated in social behavior regulation. Functional imaging studies demonstrate diminished hypothalamic activation during facial processing in interactive play scenario [136]. This pronounced reduction in hypothalamic gray matter volume, observed in children and adolescents with ASD, may underlie core social interaction deficits characteristic of the disorder. Notably, overall brain volume often remains preserved, suggesting that the specific atrophy of the hypothalamus supports the hypothesis that disruptions in neuroendocrine systems contribute to the pathophysiology of autism [137].

The hypothalamus plays a pivotal role in the synthesis and release of oxytocin, a neuropeptide critically involved in sociocognitive processes such as social recognition, trust, empathy, and interpersonal bonding. Aberrations in oxytocin levels, receptor expression, or in the structural and functional integrity of the oxytocinergic system have been proposed as contributing factors to the neurodevelopmental alterations observed in ASD. Oxytocin is essential for the modulation of social recognition and attachment, domains frequently disrupted in individuals with autism. Early studies identified reduced plasma oxytocin concentrations in children with ASD, which correlate with deficits in social communication and interaction. Additionally, evidence suggests a dysfunction in oxytocin processing, marked by an increase in biologically inactive peptide forms that may impede normal oxytocinergic signaling [138].

### 6.8. Brain Stem

A growing body of evidence implicates the brainstem in the pathogenesis of ASD. Autopsy and longitudinal MRI studies reveal agenesis of the superior olivary complex, dysgenesis of facial motor nuclei, and persistent hypoplasia of brainstem structures from infancy through adolescence, pinpointing a critical fifth-week gestational window of vulnerability [139]. Complementary volumetric analyses demonstrate that while children with ASD exhibit decreased brainstem gray matter volume at baseline, longitudinal follow-up shows progressive, bilateral gray matter expansion that normalizes by mid-adolescence [140]. Histological investigations identify significantly fewer neurons in key auditory nuclei of the brainstem, findings that parallel prolonged and asymmetric auditory brainstem reflexes and atypical sound sensitivity in ASD [141]. Functional studies further document delayed auditory brainstem response latencies, autonomic dysregulation marked by reduced cardiac vagal tone and elevated heart rate, and abnormal locus coeruleus [142]. Notably, brainstem anomalies are most prevalent in younger children with ASD, suggesting delayed maturation of neural transmission pathways [143].

Animal models reinforce these human observations: valproate-exposed rodents show aberrant neuronal migration in pontine and facial nuclei, Fmr1 knockout mice exhibit superior olivary malformations, and optogenetic activation of dorsal raphe neurons in Shank3 mutants rescues social deficits [142,144]. Finally, neuropathological reviews nominate discrete precerebellar relays—including the inferior olive, pontine nuclei, and arcuate nucleus—as loci of dysfunction underlying motor control, vestibular processing, and affect regulation in ASD [145]. Together, these structural, functional, and developmental data position the brainstem as a mechanistic hub for the sensorimotor, sensory-processing, and regulatory impairments characteristic of ASD.

### 6.9. Cerebellum

The cerebellum has garnered increasing attention in the study of ASD, reflecting its expanding role beyond motor coordination to include cognitive, affective, and social domains. Emerging research suggests that this hindbrain structure is critically involved in the neurodevelopmental alterations characteristic of ASD, influencing core symptoms such as social communication deficits, restricted interests, and repetitive behaviors. Structural and functional neuroimaging studies have consistently identified cerebellar abnormalities in individuals with ASD. Volumetric analyses reveal a heterogeneous pattern: while some studies report reduced total cerebellar volume—particularly in regions such as the posterior vermis—others show enlarged volumes or no significant differences, underscoring the phenotypic variability within the ASD population and highlighting the importance of considering age, sex, and symptom severity in analyses [146,147,148].

Converging evidence supports disrupted cerebellar white matter integrity and altered connectivity with key cerebral regions involved in social cognition and executive function [149,150]. Functional MRI studies further indicate that individuals with ASD exhibit atypical cerebellar activation patterns—ranging from hypo- to hyperactivation—during tasks involving language processing, and response inhibition [151,152]. These functional disruptions suggest that the cerebellum contributes to higher-order processes such as the temporal prediction of social cues, error correction in behavior, and the modulation of sensory inputs—all of which are compromised in ASD.

Anatomically, the cerebellum is densely interconnected with multiple cerebral areas, including the prefrontal cortex, parietal lobe, and limbic structures, allowing it to modulate a broad range of behaviors affected in ASD [153]. From a developmental perspective, the cerebellum undergoes extensive maturation during early childhood, overlapping with the typical onset of ASD symptoms. Disruptions in cerebellar development—whether due to genetic mutations, prenatal insults, or environmental exposures—can impair synaptic pruning and connectivity, potentially leading to long-term functional consequences [152,154]. Moreover, post-mortem analyses have repeatedly found reduced numbers and abnormal morphology of Purkinje cells in individuals with ASD, particularly in the posterior cerebellar hemispheres and vermis [155,156]. These findings provide a cellular substrate for the macrostructural and functional abnormalities observed through imaging and emphasize the cerebellum’s crucial role in the pathophysiology of ASD.

## 7. I’m Looking Through You

The discovery of mirror neurons (MN) in the early 1990s by Rizzolatti and colleagues revolutionized our understanding of social cognition by providing a neural basis for action understanding, imitation, and empathy [157,158]. These neurons exhibit a unique functional property: they fire both when an individual performs a specific action and when they observe another performing that same action. This “mirroring” mechanism is thought to underpin a broad array of abilities in humans, including action recognition, imitation, and social behavior [159]. The mirror neuron system (MNS) is primarily localized in the parieto-frontal cortical network, encompassing regions such as the inferior frontal gyrus and the inferior parietal lobule [160]. These areas contribute to understanding others’ actions and inferring intentions, enabling rapid, automatic social comprehension. Neuroimaging studies have shown that MN are selectively activated when actions are embedded in contexts that support intention attribution, suggesting that the MNS facilitates an intuitive, non-reflective understanding of others’ behavior [161]. When this mirroring mechanism is disrupted, as is hypothesized in ASD, individuals may be forced to rely more on explicit, cognitively effortful mentalizing processes, potentially contributing to social disinterest or interpersonal difficulties [158].

The MNS also plays a proposed evolutionary role in the emergence of language and cultural learning, offering a substrate for learning through observation and interaction [162]. In humans, MN-related activity has been observed as early as infancy, suggesting that these circuits develop rapidly in response to social input and observation of self-generated movements [163]. Given this developmental plasticity, atypical early social experiences, common in ASD, could disrupt the fine-tuning of MN circuits and their associated social functions [164]. This framework underlies the Broken Mirror Hypothesis (BMH), which posits that dysfunction in the MNS contributes to the social and communicative difficulties seen in ASD [165]. The hypothesis gained initial support from behavioral observations (e.g., deficits in imitation and pretend play) and electrophysiological findings using EEG. In particular, the mu rhythm—an EEG signal suppressed during both action execution and observation—has been used as a proxy for MN activity. Studies demonstrated that while neurotypical individuals show mu suppression in both conditions, children with ASD often show suppression only during execution, but not during observation, indicating reduced mirroring [166]. Subsequent fMRI studies revealed reduced activation in some MNS areas (e.g., inferior frontal gyrus, and inferior parietal lobule) during tasks requiring the observation of emotional expressions or goal-directed hand movements in ASD individuals compared to controls [167]. Transcranial Magnetic Stimulation research further demonstrated altered motor excitability during action observation, supporting the idea of atypical MNS functioning in ASD [168].

However, more recent findings challenge the notion of a globally “broken” mirror system (Table 2). Instead, they suggest a more nuanced disruption, involving selective impairments in specific contexts or under certain cognitive loads. For example, the EP-M model [169] proposes that individuals with ASD may have intact MN activity during goal-directed action observation but show deficits in mimicry-based imitation. The STORM model [170] highlights dysfunction in top-down modulation of the MNS by prefrontal control systems, rather than damage to the MN themselves. These models shift the explanatory emphasis from structural deficits to dynamic network dysregulation, involving aberrant connectivity and integration across distributed neural systems. Therapeutically, this evolving view has significant implications. Interventions that incorporate imitation, social reciprocity, and motor coordination, such as reciprocal imitation training or video modeling, may leverage residual MNS capacities to improve social functioning [171]. Such strategies provide indirect evidence that engaging MN-related systems can facilitate improvements in communication and social engagement in individuals with ASD. Neuroplasticity studies further support the potential for compensatory mechanisms, especially if interventions begin during critical developmental windows [172].

While the MNS may not be the sole cause of social deficits in ASD, it is still considered a crucial element within a larger network of social brain regions. The understanding in this field has evolved beyond the initial BMH. Current models emphasize the interplay between bottom-up sensory-motor processes and top-down cognitive control. Future research should focus on multimodal approaches, integrating neuroimaging, neurophysiology, and behavioral studies, to understand the contribution of MN to social cognition within this intricate neural framework.

## 8. I Should Have Known Better

Animal models have become indispensable in autism research, providing unique access to the biological underpinnings of this complex and heterogeneous condition. Given the ethical, logistical, and biological constraints inherent in studying early neurodevelopmental mechanisms directly in humans, animal models offer an irreplaceable experimental platform for hypothesis-driven research. They allow the manipulation of genetic and environmental variables under controlled conditions, making it possible to dissect the causal roles of specific risk factors and to observe how these factors interact across critical developmental windows [173]. Through these models, researchers can explore the impact of genes, toxins, immune activation, and pharmacological agents on brain structure, synaptic plasticity, neural circuitry, and behavior, hallmarks relevant to autism pathophysiology.

One of the key strengths of animal models lies in their ability to replicate conserved biological processes that are difficult or impossible to study in humans. For instance, mouse models have demonstrated how mutations in high-confidence ASD genes like *Shank3*, *Fmr1*, and *Mecp2* lead to synaptic dysregulation, abnormal dendritic spine morphology, and disrupted excitation/inhibition (E/I) balance, mechanisms implicated across multiple ASD subtypes [174,175]. Similarly, environmentally induced models, such as those using prenatal exposure to valproic acid (VPA) or maternal immune activation (MIA), reproduce features like impaired sociability, increased repetitive behaviors, and neuroinflammatory responses, offering insight into how non-genetic factors contribute to ASD phenotypes [102,176].

The diversity of animal models has expanded well beyond traditional rodents. Non-human primates (e.g., macaques and marmosets), due to their high degree of genetic, neuroanatomical, and behavioral homology with humans, are particularly valuable for studying complex social behaviors and higher-order cognitive processes. For instance, CRISPR-mediated mutations in *SHANK3* and *Mecp2* have recapitulated key ASD-related symptoms such as stereotypic movements, impaired social interaction, and altered sleep-wake cycles [177,178,179]. However, ethical concerns, long gestational periods, and high maintenance costs restrict their widespread use. Other species, such as zebrafish, songbirds, and *Drosophila*, have been harnessed to examine specific ASD-related processes. Zebrafish are useful for developmental studies due to their transparency and rapid embryogenesis, and about 70% of their genes have human orthologs [180,181]. *Drosophila* models have provided critical insights into synaptic vesicle cycling, cytoskeletal organization, and mitochondrial function in ASD, leveraging powerful genetic tools and short life cycles [182]. Songbirds, which exhibit vocal learning, have been explored to study mechanisms of speech and communication deficits, core features of ASD that are otherwise difficult to model [183].

In addition to advancing basic understanding, animal models play a pivotal role in identifying convergent biological pathways. These convergences suggest that therapeutic interventions targeting shared downstream pathways may have broader efficacy across ASD subtypes. Therapeutic advances inspired by animal studies include not only pharmacological agents but also non-drug interventions. Environmental enrichment, characterized by enhanced sensory and social stimulation, has been shown to mitigate behavioral abnormalities in multiple ASD models by promoting neurogenesis, enhancing synaptic plasticity, and modulating behavior [184,185]. These findings underscore the value of integrating biological and experiential interventions, and support early behavioral therapies in clinical contexts.

Nevertheless, important limitations must be acknowledged. No model can fully replicate the human autism phenotype, particularly given the roles of culture, language, and subjective experience. There is also a translational gap: many drugs effective in animal models have failed in human trials, underscoring the need for better biomarkers and more sophisticated behavioral phenotyping. To overcome this, the field is moving toward automated tracking, machine learning for behavioral classification, and standardized cross-species testing paradigms. In hindsight, perhaps we truly “should have known better”: understanding autism demands more than clinical observation, it demands experimental insight into developmental biology, circuit physiology, and gene-environment interactions. As the field evolves, animal models remain a cornerstone of ASD research, because they reflect its essential mechanisms with clarity and precision.

## 9. With a Little Help from My Friends

While the precise etiology of ASD remains multifactorial and not fully understood, growing evidence highlights the involvement of neurotrophins—a family of proteins essential for the survival, development, and function of neurons. Neurotrophins, including Brain-Derived Neurotrophic Factor (BDNF), Nerve Growth Factor (NGF), Neurotrophin-3 (NT-3), and Neurotrophin-4/5 (NT-4/5), play pivotal roles across the developing and mature nervous system [186]. Their functions encompass: Neuronal survival and differentiation—ensuring the viability of developing neurons and guiding them to mature forms; axonal and dendritic growth—promoting the extension and branching necessary for neural circuit formation; and, synaptogenesis and synaptic plasticity—regulating the formation and modification of synapses [187].

Given their central role in brain wiring and plasticity, alterations in neurotrophin levels or receptor signaling during critical developmental periods have been hypothesized to contribute to the atypical neural connectivity observed in ASD. Among neurotrophins, BDNF has been the most extensively studied. Meta-analyses suggest that, as a group, individuals with ASD may exhibit higher peripheral BDNF levels compared to neurotypical controls. Yet findings remain inconsistent, with reports of lower or unchanged levels in some studies. These discrepancies likely reflect the heterogeneity of ASD itself, along with methodological factors such as age, sex, sample type (serum vs. plasma), and analytical techniques [188,189].

The biological effects of neurotrophins are mediated primarily through two receptor families: Trk receptors (TrkA, TrkB, TrkC), which activate signaling cascades that promote neuronal survival, growth, and synaptic plasticity; and the p75 neurotrophin receptor (p75NTR), which can promote either survival or apoptosis depending on cellular context and co-receptor interactions. The delicate balance between Trk- and p75NTR-mediated signaling is therefore critical for normal neurodevelopment. Dysregulation of this balance has been proposed as a contributor to ASD pathophysiology [188].

Animal models have been particularly informative in probing these mechanisms. Rodent studies demonstrate that both increased and decreased BDNF expression during sensitive developmental windows can produce behavioral phenotypes reminiscent of ASD. Elevated BDNF may lead to altered social behavior and heightened repetitive actions, whereas reduced BDNF signaling has been linked to impairments in social interaction and cognitive flexibility. These contradictory findings mirror the inconsistencies in human studies and underscore that the timing, location, and regulation of neurotrophin expression are as critical as their overall levels [190,191].

Taken together, the evidence suggests that neurotrophins represent an important, though complex, piece of the autism puzzle. Their dual roles in neuronal survival and plasticity, and their variable expression across contexts, highlight the need for further investigation into how these molecules shape the developmental trajectory of the autistic brain.

## 10. Savoy Truffle

Among the environmental factors implicated in ASD, the gut microbiota has emerged as a particularly compelling area of investigation. This complex ecosystem of microorganisms in the gastrointestinal tract regulates not only digestion and metabolism but also immune function and neural development through the gut–brain axis. This bidirectional communication system, which involves the central nervous system (CNS), autonomic nervous system (ANS), enteric nervous system (ENS), and the hypothalamic–pituitary–adrenal (HPA) axis, provides a mechanistic link through which the gut microbiota can influence brain function. The vagus nerve, a key component of the parasympathetic nervous system, serves as a direct conduit between gut and brain, transmitting signals modulated by microbial metabolites [192].

Gastrointestinal symptoms such as constipation, diarrhea, and abdominal pain occur with higher prevalence in individuals with ASD, fueling the hypothesis that microbiota dysbiosis—an imbalance in microbial communities—may contribute to both gastrointestinal and behavioral phenotypes. Dysbiosis can alter immune regulation, induce chronic inflammation, and shift the production of metabolites such as short-chain fatty acids (SCFAs), neurotransmitters (serotonin, dopamine), and other neuroactive compounds. Research on the gut microbiota in ASD, though not always consistent, has identified common patterns. Some studies report reduced microbial diversity, while others describe altered ratios of the dominant bacterial phyla (Firmicutes and Bacteroidetes) or shifts in key genera including *Lactobacillus*, *Bifidobacterium*, *Prevotella*, and *Clostridium*. Such alterations may be compounded by the highly selective and restricted diets common in ASD, which are often low in fiber and high in processed foods, reducing beneficial SCFA-producing bacteria [192,193].

Given these findings, dietary interventions aimed at modulating the microbiota have gained increasing attention [194,195]:

Probiotics and Prebiotics: Probiotics introduce beneficial bacteria such as *Lactobacillus* and *Bifidobacterium*, improving gut barrier function and reducing inflammation. Prebiotics (e.g., inulin, fructo-oligosaccharides) nourish endogenous microbes, enhancing SCFA production and modulating the gut–brain axis. Some studies report improvements in both gastrointestinal and behavioral symptoms.

Fecal Microbiota Transplantation (FMT): Also known as microbiota transfer therapy, FMT seeks to restore microbial diversity by transferring fecal material from healthy donors. Early studies in ASD suggest long-lasting improvements in both gastrointestinal and core behavioral symptoms, accompanied by increased microbial diversity and altered metabolite profiles, making the recipient’s microbiota more similar to that of neurotypical individuals.

Gluten-Free and Casein-Free (GFCF) Diets: Although widely used, evidence for GFCF diets remains mixed. The “opioid excess” theory proposes that incompletely digested peptides from gluten and casein may cross a “leaky gut” barrier, reach the brain, and interfere with neurotransmission. Removal of these proteins may also reduce inflammation in sensitive individuals.

Altogether, the gut microbiota and diet are emerging as critical players in ASD pathophysiology. While current evidence supports their potential influence on both gastrointestinal and behavioral dimensions, the field remains marked by inconsistencies. Future research must refine our understanding of how microbiota–diet interactions shape brain development and behavior, and how this knowledge can guide personalized, targeted interventions for individuals across the autism spectrum.

## 11. Golden Slumbers

This is the end of this journey, where we are reminded that the road to understanding autism is both a scientific and human endeavor, one marked by complexity, resilience, and hope. Like the lullaby in *Golden Slumbers*, this final note is not a conclusion, but a pause in a long and evolving narrative. We should not rest in complacency, but reflect critically on where the science of autism stands and where it must go. The metaphor is fitting: after the rigors of exploration, a moment of calm is necessary to integrate what has been learned and to envision the next phase, not just in research, but in the ethical, clinical, and policy landscapes that surround ASD.

Throughout this work, we have traversed the intricate landscapes of neural development, genetic pathways, brain circuitry, animal models, and socio-emotional functions. Each section has revealed not only the remarkable progress in autism research but also the persistent gaps that invite further exploration. The analogies drawn from The Beatles’ discography have served as more than literary devices; they reflect the rhythm, emotion, and philosophical undercurrents that run parallel to our scientific inquiries. ASD continues to challenge our categories, our diagnostics, and even our language. Yet, it also pushes the frontiers of neuroscience, behavioral science, and inclusive thought. In embracing its heterogeneity, we are called to move beyond simple explanations and seek integrative, nuanced models that honor both biological diversity and individual experience.

Let this slumber be a brief rest before the next phase of questioning, experimenting, and listening. For, in the words that follow Golden Slumbers, “Carry that weight” reminds us that the responsibility to understand, to include, and to act remains with us all. Autism, in its complexity and heterogeneity, challenges our traditional frameworks of diagnosis, intervention, and inclusion. It has compelled science to evolve from categorical models to dimensional ones, from isolated brain region theories to dynamic network models, and from simplistic genetic explanations to multi-layered systems biology. We now understand that ASD arises from a confluence of factors: genetic, epigenetic, environmental, developmental, and socio-cultural. And yet, this understanding, as rich as it is, remains unevenly translated into practice.

Scientific advances have outpaced public health infrastructure. While imaging, molecular biology, and behavioral sciences have made extraordinary strides, early diagnosis is still delayed in many regions. Access to evidence-based interventions remains limited and fragmented. Support across the lifespan, particularly during adolescence, adulthood, and aging, is often an afterthought in national health agendas. Families shoulder the weight of navigation in systems that are frequently underfunded, uncoordinated, and inequitable.

Animal models, neuroimaging, and genetics have illuminated important mechanisms underlying ASD, but policy must now ensure that these insights inform real-world decision-making. Investments are needed not only in basic and translational research, but in longitudinal studies, implementation science, and community-based participatory research. Educational systems must be equipped to respond to diverse learning needs; healthcare providers must be trained in neurodiversity-informed care; and research funding bodies must prioritize interdisciplinary and inclusive approaches. Furthermore, ethical leadership is essential. The future of autism research must be guided by the voices of autistic individuals themselves, as partners in shaping priorities, interpreting findings, and defining success. This demands a cultural shift in academia and policy circles: from studying autism to co-creating knowledge with the autism community. It also requires that we stop treating inclusion as an endpoint and start recognizing it as a continuous process of structural change.

Autism Spectrum Disorder is not merely a biomedical science challenge; it is a societal question that intersects with education, labor, justice, health, and human rights. Let us ensure that the next chapter of autism science is written not only with data, but with vision, inclusivity, and concrete action. This requires advancing diagnostic protocols that better account for sex differences and developmental trajectories, prioritizing funding for interdisciplinary and community-based research, and fostering policies that actively include the voices of autistic individuals and their families. And so, as this review ends, we return to the song that lent this manuscript its title. The Long and Winding Road still stretches ahead, unpredictable, but full of potential. It reminds us that science is not a straight path to knowledge, but a journey shaped by empathy, uncertainty, and the courage to listen. We walk it not alone, but together, with researchers, clinicians, families, and autistic individuals alike, carrying the weight, and the hope, that each step brings us closer to understanding.

## Figures and Tables

**Table 1:** Brain Areas Affected in Autism Spectrum Disorder (ASD).

Brain Area	General Role	Relevance in ASD
Cerebral Cortex	High order cognitive processes	Abnormal folding and altered connectivity
Basal Ganglia	Motor control, higher-order cognition, speech	Volumetric changes and reduced connectivity
Amygdala	Emotion regulation, fear processing, social salience	Enlargement and hypoactivation
Hippocampus	Memory formation, reasoning, social interaction	Structural differences and altered connectivity
Thalamus	Relay center of connectivity between cortical and subcortical regions	Atypical neural connectivity
Hypothalamus	Neuroendocrine regulation of social behavior	Reduced neuronal density and alterations in the oxytocinergic system
Brainstem	Sensory processing and autonomic regulation	Reduced volume, abnormal auditory processing, and autonomic dysregulation
Cerebellum	Motor coordination, cognitive and social modulation	Reduced and abnormal Purkinje neurons, with structural and functional anomalies

**Table 2:** Comparative Models of Mirror Function in Autism Spectrum Disorder (ASD).

Model	Core Assumption	Relevance in ASD
Broken Mirror Hypothesis	Dysfunction of the mirror neuron system (MNS) leads to social and communicative alterations	Initially influential in linking neural activity to autism-related social deficits
EP-M Model (Emulation–Prediction Model)	The MNS is intact in ASD; but deficits occur in prediction and mimicry-based imitation	Provides a more dynamic account of action understanding, linking MNS activity to broader predictive processing systems
STORM Model (Social Top-Down Response Modulation)	The MNS is intact in ASD; impairments arise from atypical top-down modulation of MNS by prefrontal control systems	Suggests that social difficulties result from impaired connectivity rather than a broken MNS

## Data Availability

Not applicable.
